# The crosstalk between glomerular endothelial cells and podocytes controls their responses to metabolic stimuli in diabetic nephropathy

**DOI:** 10.1038/s41598-023-45139-7

**Published:** 2023-10-20

**Authors:** Michael Albrecht, Carsten Sticht, Tabea Wagner, Steffen A. Hettler, Carolina De La Torre, Jiedong Qiu, Norbert Gretz, Thomas Albrecht, Benito Yard, Jonathan P. Sleeman, Boyan K. Garvalov

**Affiliations:** 1 European Center for Angioscience (ECAS), Medical Faculty Mannheim of the University of Heidelberg, Ludolf-Krehl-Strasse 13–17, 68167 Mannheim, Germany; 2 Mannheim Institute for Innate Immunoscience (MI3), Medical Faculty Mannheim of the University of Heidelberg, Ludolf-Krehl-Strasse 13–17, 68167 Mannheim, Germany; 3 Center of Medical Research, Bioinformatics and Statistics, Medical Faculty Mannheim of the University of Heidelberg, Theodor-Kutzer-Ufer 1-3, 68167 Mannheim, Germany; 4 NGS Core Facility, Medical Faculty Mannheim of the University of Heidelberg, Theodor-Kutzer-Ufer 1-3, 68167 Mannheim, Germany; 5 Department of Nephrology, Hypertensiology, Endocrinology, Diabetology, Rheumatology and Pneumology, Fifth Department of Medicine, Medical Faculty Mannheim of the University of Heidelberg, Mannheim, Germany; 6 Institute of Pathology, University Hospital Heidelberg, Im Neuenheimer Feld 224, Heidelberg, Germany; 7 Institute of Biological and Chemical Systems – Biological Information Processing (IBCS-BIP), Karlsruhe Institute of Technology Campus North, Building 319, Hermann-Von-Helmholtz-Platz 1, 76344 Eggenstein-Leopoldshafen, Germany

**Keywords:** Diabetes complications, Diabetic nephropathy, Transcriptomics, Mechanisms of disease

## Abstract

In diabetic nephropathy (DN), glomerular endothelial cells (GECs) and podocytes undergo pathological alterations, which are influenced by metabolic changes characteristic of diabetes, including hyperglycaemia (HG) and elevated methylglyoxal (MGO) levels. However, it remains insufficiently understood what effects these metabolic factors have on GEC and podocytes and to what extent the interactions between the two cell types can modulate these effects. To address these questions, we established a co-culture system in which GECs and podocytes were grown together in close proximity, and assessed transcriptional changes in each cell type after exposure to HG and MGO. We found that HG and MGO had distinct effects on gene expression and that the effect of each treatment was markedly different between GECs and podocytes. HG treatment led to upregulation of “immediate early response” genes, particularly those of the EGR family, as well as genes involved in inflammatory responses (in GECs) or DNA replication/cell cycle (in podocytes). Interestingly, both HG and MGO led to downregulation of genes related to extracellular matrix organisation in podocytes. Crucially, the transcriptional responses of GECs and podocytes were dependent on their interaction with each other, as many of the prominently regulated genes in co-culture of the two cell types were not significantly changed when monocultures of the cells were exposed to the same stimuli. Finally, the changes in the expression of selected genes were validated in BTBR *ob/ob* mice, an established model of DN. This work highlights the molecular alterations in GECs and podocytes in response to the key diabetic metabolic triggers HG and MGO, as well as the central role of GEC-podocyte crosstalk in governing these responses.

## Introduction

Diabetic nephropathy (DN) develops in around one third of patients with diabetes and represents one of the principal complications of the disease^1^. DN involves progressive loss of kidney function, often leading to end-stage renal disease that requires kidney replacement therapy. As a result, diabetes has become the leading primary cause of chronic kidney disease and end-stage renal disease, which are associated with considerable morbidity, mortality and economic burden^2^. Current therapeutic options for DN are limited and include strict control of blood glucose or lowering of blood pressure. However, both the diagnosis and treatment of early stage DN remain challenging, in part due to insufficient understanding of the molecular mechanisms involved in DN pathogenesis.

Hyperglycaemia (HG) is a major risk factor for DN, and is thought to drive the early pathological changes in DN as intensive glycaemic control is associated with a marked decrease in the incidence of diabetic nephropathy in both type 1 and type 2 diabetes patients^3^. Elevated glucose levels in diabetic patients are linked to the increased formation of dicarbonyl compounds, in particular methylglyoxal (MGO)^4–6^, which is a by-product of glycolysis. MGO is a potent glycating agent that plays a key role in the generation of advanced glycation end products (AGEs) in diabetic patients^7–9^. AGEs, which include modified proteins, lipids and DNA, can affect multiple biological processes, and the accumulation of MGO-derived AGEs correlates with early progression in DN^7^. Evidence for a potential causative role of MGO in DN pathology has been provided by studies using mice lacking the MGO-detoxifying enzyme glyoxalase 1, which had increased MGO levels and developed renal alterations similar to those caused by diabetes^10^.

DN is characterised by compromised function of the glomerular filtration barrier. The key cell types that make up this barrier are glomerular endothelial cells (GECs), which line the luminal surface of the glomerular capillaries, and podocytes, a specialised type of epithelial cells that share a common basement membrane with endothelial cells and tightly wrap around it. Impaired function of both cells types is one of the primary events during the early stages of DN^11–13^. GECs and podocytes are involved in a complex and intimate crosstalk with each other to ensure the normal filtration of the blood, e.g. via diverse growth factors, chemokines and other secreted molecules, which can be altered in DN^14,15^. Recent work has shown that partial podocyte depletion in vivo leads to profound changes in the expression profile of GECs and pathological alterations^16^, indicating that the crosstalk between these cells types is essential for their normal function and the response to pathogenic triggers. Transcriptomic analyses of GECs^17–21^ and podocytes^18,19,21–24^ from preclinical models of DN or DN patients have provided important insights into the alterations of these cells in diabetic conditions. However, the specific contribution of individual DN risk factors, such as HG and MGO, to changes in GECs and podocytes remains insufficiently understood. Moreover, little is known about the potential influence that GECs and podocytes can have on each other when exposed to such stimuli, and how this could affect their transcriptional responses.

To address these questions, we established a co-culture system in which conditionally immortalised human GECs and podocytes were grown in close proximity, allowing communication between the cells through secreted factors or vesicles. The cells were then exposed to HG or MGO and transcriptomic changes in each cell type were analysed by RNA sequencing. We found that these stimuli had very different effects on the two cells types with little overlap of differentially expressed genes (DEGs) between HG and MGO-treated cells. In both GECs and podocytes, HG, but not MGO, led to consistent upregulation of immediate early response genes. We could validate the differential expression of numerous genes in GEC/podocyte co-cultures, but crucially, none of the examined genes showed changed expression in monocultures of the respective cell type exposed to HG or MGO, demonstrating that the crosstalk between GECs and podocytes plays a crucial role in determining their response to DN-relevant metabolic stimuli. The altered regulation of selected genes from our analyses was confirmed in kidneys from the BTBR *ob/ob* mouse model of DN.

## Results

### Establishment of a GEC-podocyte co-culture system for analysis of gene expression

**Figure 1:**
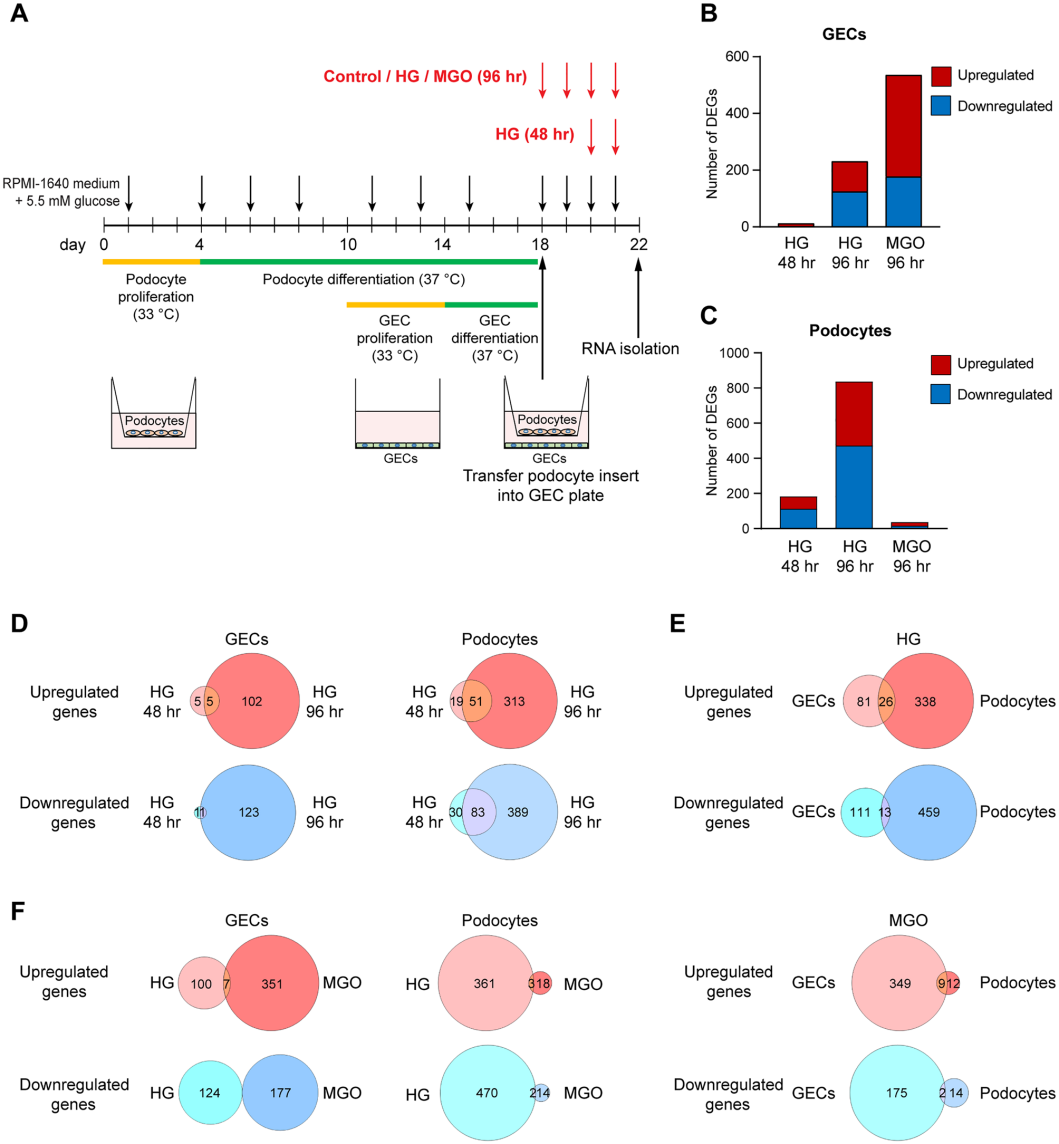
Experimental setup and overview of the differentially expressed genes after hyperglycaemia (HG) or methylglyoxal treatment (MGO). **(A)** Schematic diagram of the GEC/podocyte co-culture and HG or MGO treatment. Podocytes were seeded on the membrane of a transwell insert and glomerular endothelial cells (GECs) were seeded on the bottom of the transwell. Both cell types were differentiated in such a way that the end of their differentiation phases (14 days for podocytes and 4 days for GECs) coincided. At the end of the differentiation phase, the podocytes and GECs were brought together in the same plate and treated with 25 mM glucose (HG, addition of 19.5 mM glucose to RPMI-1640 medium containing 5.5 mM glucose) for 48 h or 96 h, or with 200 µM MGO for 96 h. 5.5 mM glucose + 19.5 mM mannitol (osmotic control) and water served as treatment controls, respectively. Downward facing arrows indicate medium change/refreshment; during stimulation, medium was refreshed each day. At the end of the experiment, RNA was isolated separately from each cell type and analysed by RNA-seq. (**B**,**C**) Total numbers of genes that were significantly differentially expressed in GECs (**B**) and podocytes (**C**) after the different treatments. DEGs: differentially expressed genes. (**D–F**) Venn diagrams showing the overlaps of up- and downregulated genes between 48 and 96 h of HG stimulation for each cell type (**D**), between podocytes and GECs for 96 h HG and MGO (**E**) and between 96 h HG and MGO for each cell type (**F**). Upregulated genes sets are represented by red hues, downregulated gene sets by blue hues.

To assess the crosstalk between GECs and podocytes under conditions of hyperglycaemia and increased MGO levels, we set up a transwell coculture system with human cells that were conditionally immortalised by expression of temperature sensitive SV40 large T antigen. These cells are well-established models for studying GEC and podocyte biology in cell culture^25–27^, and proliferate at the permissive temperature of 33 °C, at which the large T antigen is active. Upon a switch to 37 °C, the large T antigen is inactivated, proliferation ceases and the cells regain their differentiated states. Since podocytes and GECs require different times for their differentiation, we first seeded conditionally immortalised podocytes in a transwell insert with a membrane containing 0.4 µm-sized pores that allow exchange of soluble factors and small extracellular vesicles, but no direct mixing of cells. After a 4-day proliferation phase at 33 °C, the podocytes were differentiated for 14 days at 37 °C (Fig. 1A). During this period, conditionally immortalised GECs were seeded at the bottom of the transwells and grown at 33 °C for 4 days followed by differentiation at 37 °C for additional 4 days, timed so as to coincide with the end of podocyte differentiation (Fig. 1A). To study the effects of HG on the co-cultured cells, the transwell inserts carrying the podocytes were then placed on top of the wells with the GECs, and the cells were subsequently cultured in the presence of 25 mM glucose (HG) for 48 or 96 h. Medium with normal (5.5. mM) glucose + 19.5 mM mannitol (to control for osmotic effects) served as control. The effects of methylglyoxal were studied following culture in the presence of 200 µM MGO for 96 h (Fig. 1A). At the end of the experiments, RNA was isolated separately from the GECs at the bottom of the well and the podocytes in the insert, and gene expression was analysed by RNA-seq. Neither HG nor MGO treatment had a significant impact on the number of GECs or podocytes at the endpoint (Fig. S1A–D).

### Global effects of HG and MGO on gene expression in GECs and podocytes

In GECs, few genes were differentially expressed after 48 h of HG, but the number of DEGs increased substantially at 96 h. Treatment of GECs with MGO induced differential expression of the largest number of genes (Fig. 1B, Supplementary Table S1). Podocytes responded differently to these treatments. Hyperglycaemia led to considerably greater changes in gene expression already after 48 h, which were further enhanced after 96 h. By contrast, exposure to MGO had a rather limited effect on gene expression (Fig. 1C, Supplementary Table S1). These results indicate that GECs and podocytes respond very differently to metabolic challenges typical for diabetes, such as HG and MGO.

Comparison of the expression patterns at different time points of HG revealed that most of the genes that were regulated at 48 h remained differentially expressed at 96 h, but a large number of additional genes were up- and downregulated after 96 h in both cell types (Fig. 1D, Supplementary Table S2). The DEGs in GECs and podocytes, however, were mostly distinct from each other, with limited overlap between the two cells types following exposure to either HG or MGO (Fig. 1E, Supplementary Table S2). In addition, the effects of HG and MGO on both cell types were very distinct with no or very little overlap between the two conditions (Fig. 1F, Supplementary Table S2).

### HG regulates genes involved in the immediate early response and inflammatory responses in GECs co-cultured with podocytes

**Figure 2:**
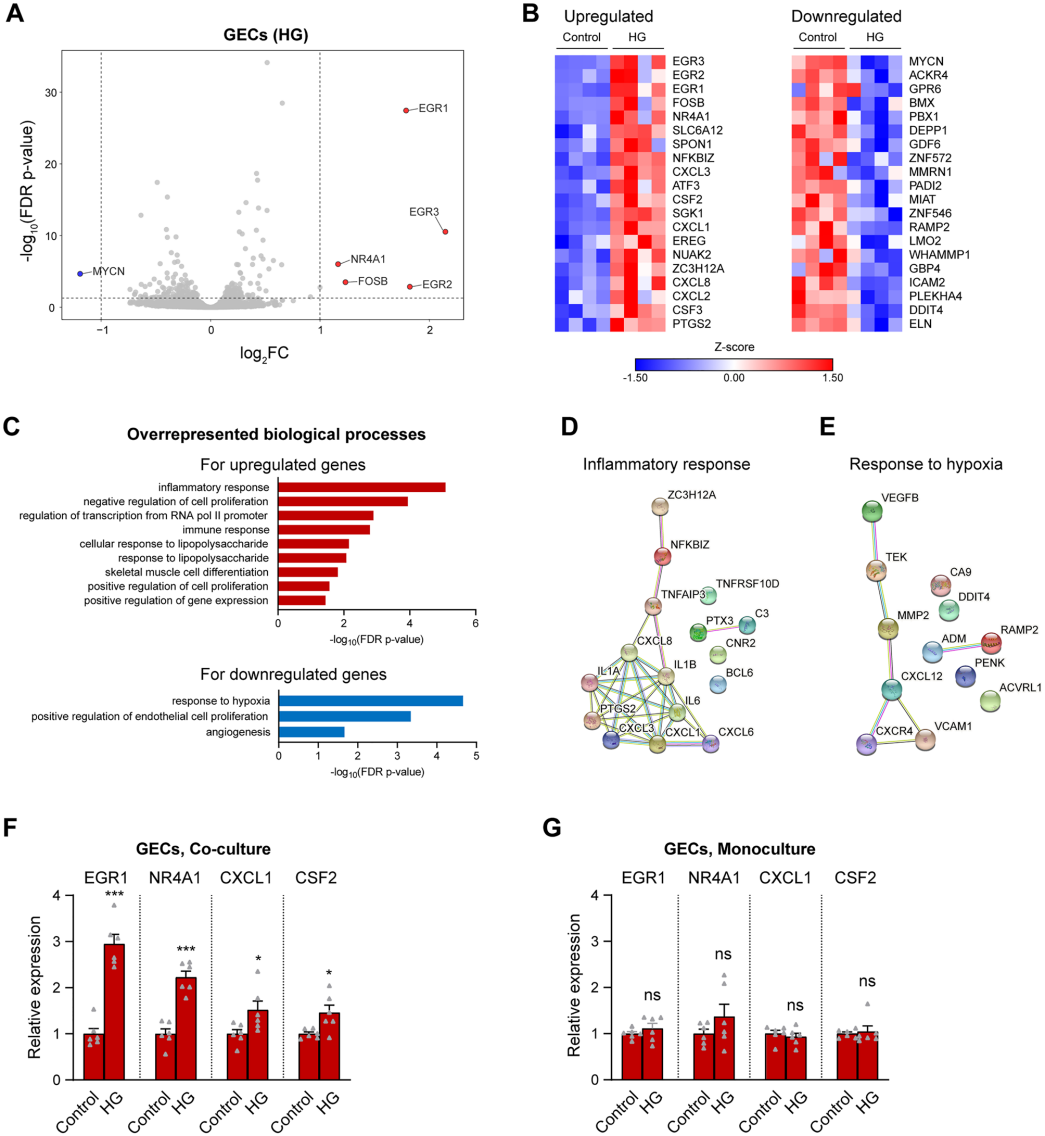
Hyperglycaemia upregulates inflammatory genes and downregulates genes linked to the hypoxic response in GECs co-cultured with podocytes. (**A**) Volcano plot of gene expression changes between GECs exposed to 96 h of HG and control GECs. All genes above the dotted horizontal line were significantly differentially expressed (false discovery rate [FDR]-corrected p-value < 0.05). Genes with log_2_ fold change (log_2_FC) > 1 and FDR-corrected p-values < 0.05 are marked in red (strongly upregulated genes, to the right of the vertical dotted lines); genes with log_2_FC < − 1 and FDR-corrected p-values < 0.05 are highlighted in blue (strongly downregulated genes, to the left of the vertical dotted lines). (**B**) Heat maps of the top 20 up- and downregulated genes in GECs after 96 h of HG. (**C**) Functional annotation showing overrepresented gene ontology (GO) biological processes for the upregulated (red bars) and downregulated (blue bars) genes in GECs after 96 h of HG. (**D**,**E**) Interaction network of the HG-regulated genes belonging to the top GO terms in GECs, namely “inflammatory response” for the upregulated genes (**D**), and “response to hypoxia” for the downregulated genes (**E**), generated using the STRING database, based on high-confidence interactions (interaction score ≥ 0.7). (**F**,**G**) qPCR analysis of the expression of selected candidate genes that were among the top upregulated genes in GECs after 96 h of HG, based on the RNA-seq results (EGR1, NR4A1, CXCL1, CSF2). The relative expression levels of the genes in co-cultures of GECs with podocytes (**F**) and in GEC monocultures (**G**) are shown. For both co-cultures and monocultures n = 6.

In GECs, several of the most highly regulated genes by HG belonged to the group of the “immediate early response genes”^28^, e.g. EGR1-3, FOSB and NR4A1, which were the five most highly upregulated genes (Fig. 2A,B, Supplementary Table S1). Other genes including growth factors and cytokines were also among the top upregulated genes.

Systematic functional annotation of the DEGs in GECs for gene ontology biological processes revealed several overrepresented groups. Among the most significantly overrepresented functional groups for the upregulated genes were inflammatory/immune response, negative regulation of cell proliferation, and regulation of transcription from polymerase II promoters (Fig. 2C,D). The downregulated genes were enriched for genes involved in the response to hypoxia, endothelial cell proliferation and angiogenesis (Fig. 2C,E). We next validated by qPCR the differential expression for some of the most highly upregulated genes, including EGR1, NR4A1, CXCL1 and CSF2, in independent co-cultures of GECs and podocytes. In all cases, a significant upregulation was observed after HG (Fig. 2F), replicating the RNA-seq results. Crucially however, when GECs were cultured on their own without podocytes, none of these genes was upregulated by HG (Fig. 2G), demonstrating that the interaction with podocytes is instrumental for controlling gene expression in GECs in response to HG.

### Differential expression of genes related to the cell cycle, extracellular matrix and cell adhesion in podocytes co-cultured with GECs under HG

**Figure 3:**
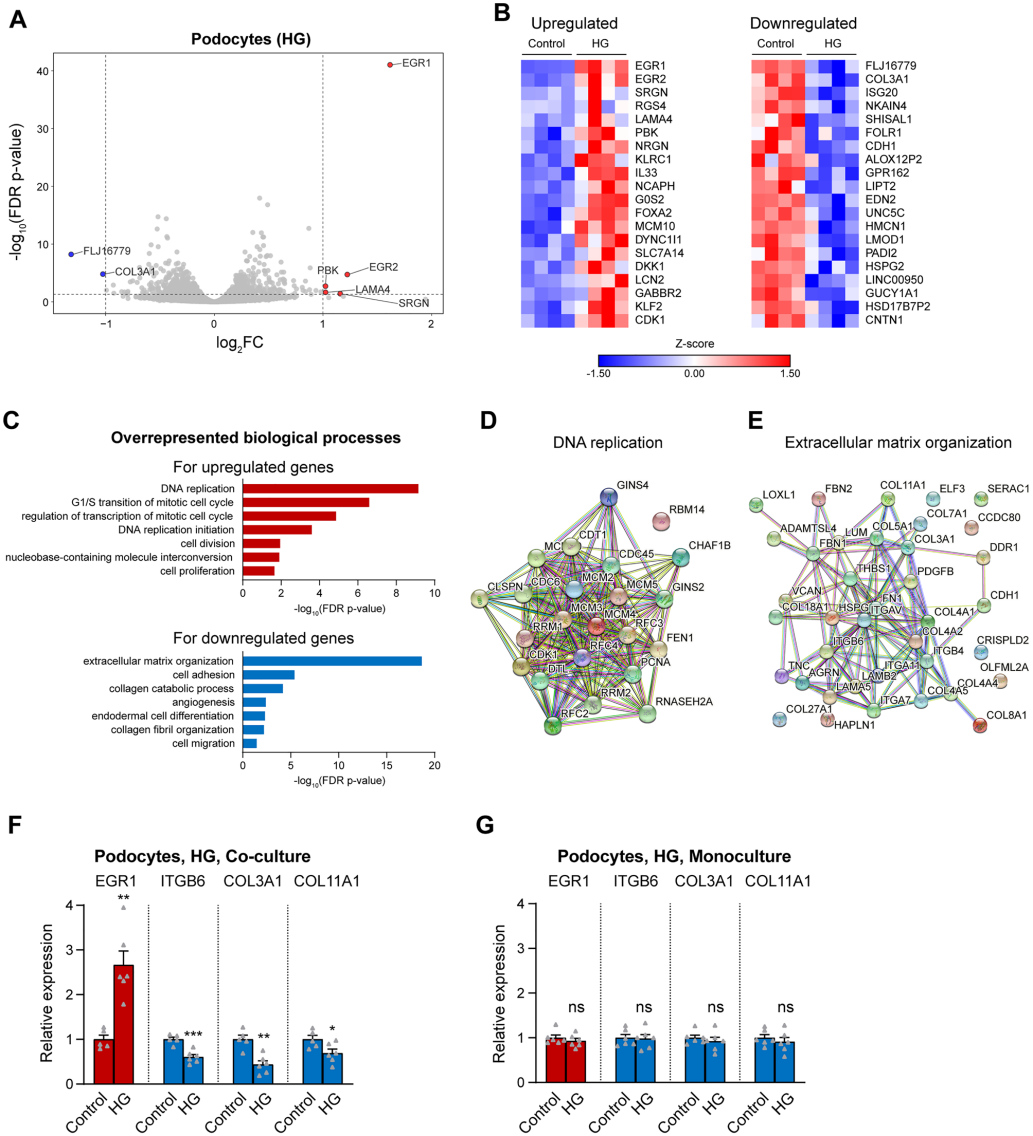
Hyperglycaemia upregulates immediate early and cell cycle genes and downregulates genes related to the ECM and cell adhesion in podocytes co-cultured with GECs. (**A**) Volcano plot of gene expression changes between podocytes exposed to 96 h of HG and control podocytes. Genes with log_2_FC > 1 and FDR-corrected p-values < 0.05 were marked in red (strongly upregulated genes); genes with log_2_FC < − 1 and FDR-corrected p-values < 0.05 were highlighted in blue (strongly downregulated genes). (**B**) Heat maps of the top 20 up- and downregulated genes in podocytes after 96 h of HG. (**C**) Functional annotation showing overrepresented gene ontology (GO) biological processes for the upregulated (red bars) and downregulated (blue bars) genes in podocytes after 96 h of HG. (**D**,**E**) Interaction network of the HG-regulated genes belonging to the top GO terms in podocytes, namely “DNA replication” for the upregulated genes (**D**) and “extracellular matrix organization” for the downregulated genes (**E**), generated using the STRING database, based on high-confidence interactions (interaction score ≥ 0.7). (**F,G**) qPCR analysis of the expression of selected candidate genes that were among the top upregulated gene (EGR1, red bars) and downregulated genes (ITGB6, COL3A1, COL11A1, blue bars) in podocytes after 96 h of HG, based on the RNA-seq results. The relative expression levels of the genes in co-cultures of podocytes with GECs (**F**) and in podocyte monocultures (**G**) are shown. n = 6, except for HG-treated podocytes in coculture, for which n = 5.

In podocytes, the immediate early response genes EGR1 and EGR2 were the top upregulated genes following HG, similar to the case in GECs (Fig. 3A,B, Supplementary Table S1). However, most of the remaining DE genes were distinct from those in GECs (Supplementary Table S1, Fig. 1E). Gene ontology analysis demonstrated overrepresentation of several functional groups linked to the cell cycle and DNA replication among the upregulated genes in podocytes (Fig. 3C,D). The downregulated gene set, on the other hand, was enriched for genes involved in extracellular matrix (ECM) organisation, cell adhesion and cell migration (Fig. 3C,E). We further validated by qPCR the upregulation of EGR1, as well as the downregulation of several ECM components/receptors, including ITGB6, COL3A1 and COL11A1 in independent samples from podocytes co-cultured with GECs (Fig. 3F). As was the case for GECs, co-culture was required for eliciting the observed gene expression changes in podocytes, since in podocyte monocultures that did not include GECs, none of the above-mentioned genes was altered (Fig. 3G).

### MGO has little effect on podocytes but induces a distinct shift in the GEC transcriptome

**Figure 4:**
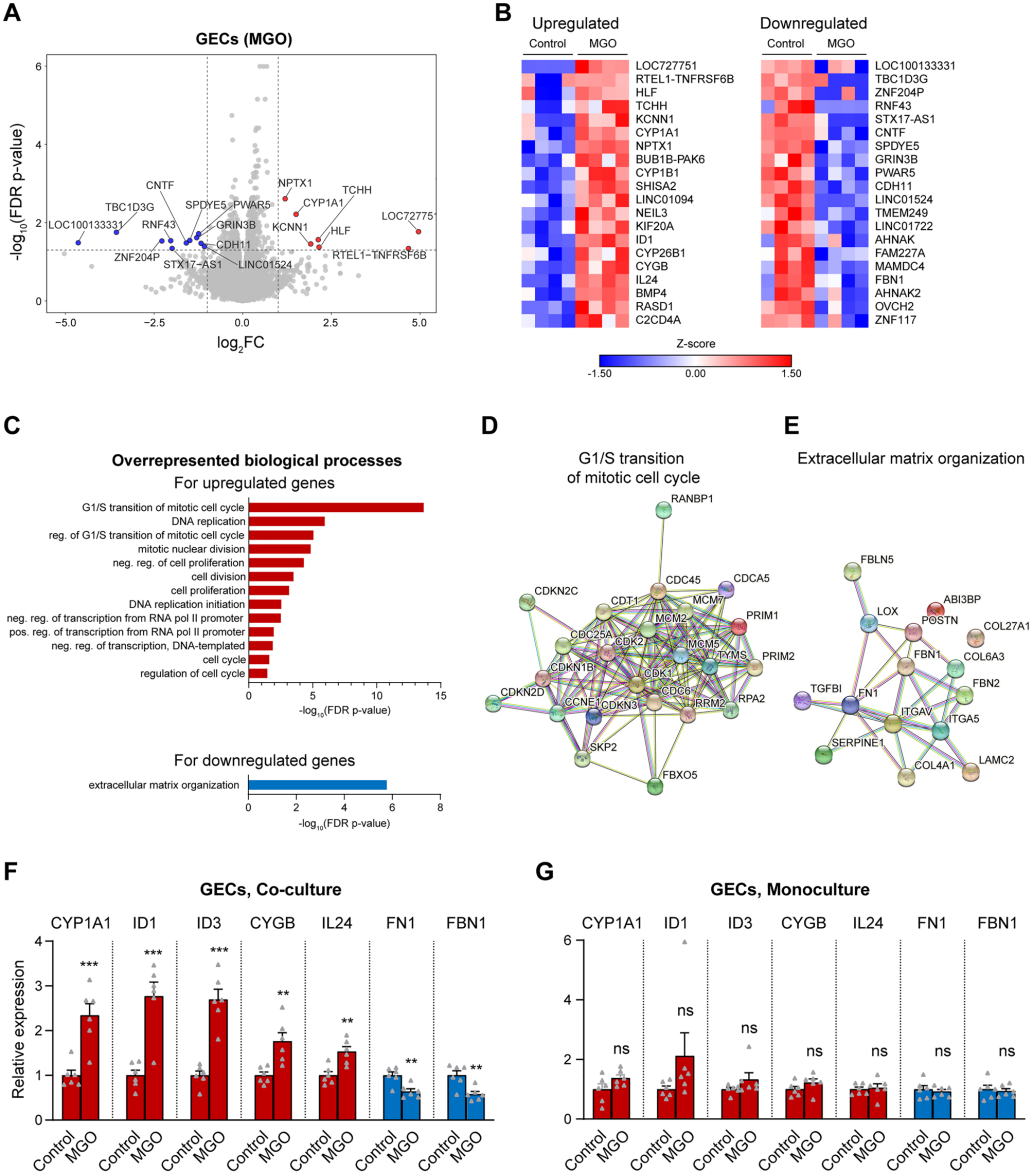
MGO upregulates cell cycle genes and downregulates genes related to ECM organisation in GECs co-cultured with podocytes. (**A**) Volcano plot of gene expression changes in GECs exposed to 96 h of MGO compared to control cells. Genes with log_2_FC > 1 and FDR-corrected p-values < 0.05 were marked in red (strongly upregulated genes); genes with log_2_FC < − 1 and FDR-corrected p-values < 0.05 were highlighted in blue (strongly downregulated genes). (**B**) Heatmaps of the top 20 up- and downregulated genes in GECs after 96 h of MGO. (**C**) Functional annotation showing overrepresented gene ontology (GO) biological processes for the upregulated (red bars) and downregulated (blue bars) genes in GECs after 96 h of HG. Abbreviations used: neg., negative; pos., positive; reg., regulation; reg. of G1/S transition of mitotic cell cycle, regulation of transcription involved in G1/S transition of mitotic cell cycle. (**D**,**E**) Interaction network of the MGO-regulated genes belonging to the top GO terms in GECs, namely “G1/S transition of mitotic cell cycle” for the upregulated genes (**D**) and “extracellular matrix organization” for the downregulated genes (**E**), generated using the STRING database, based on high-confidence interactions (interaction score ≥ 0.7). (**F,G**) qPCR analysis of the expression of selected candidate genes that were among the prominent upregulated genes (CYP1A1, ID1, ID3, CYGB, IL24, red bars) and the prominent downregulated genes (FN1, FBN1, blue bars) in GECs after 96 h of MGO, based on the RNA-seq results. The relative expression levels of the genes in co-cultures of GECs with podocytes (**F**) and in GEC monocultures (**G**) are shown. For both co-cultures and monocultures n = 6.

We next assessed the changes induced by exposure of co-cultured GEC and podocytes to MGO. In podocytes, MGO had a surprisingly limited effect, with less than 40 genes showing a significant change in expression (Fig. S2A, B). This was in contrast to the marked changes seen in GECs under the same conditions, in which over 500 genes were differentially expressed (Fig. 4A, see also Fig. 1B). No functional groups were overrepresented among the few up- and downregulated genes in podocytes. In GECs, MGO treatment led to upregulation of genes linked to the cell cycle and downregulation of genes involved in ECM organisation (Fig. 4C–E). While these functional groups were similar to the ones that were altered by HG in podocytes (Fig. 3C), the specific genes affected by HG in podocytes and by MGO in GECs were largely distinct (Fig. S3, Supplementary Table S1).

We next validated by qPCR the changes in the expression of selected up- and downregulated genes following MGO treatment (Fig. 4B, Supplementary Table S1) in independent samples of GECs co-cultured with podocytes. These results confirmed the changes in the expression of multiple genes including CYP1A1, ID1, ID3, CYGB, IL24, FN1 and FBN1 (Fig. 4F). Importantly, as seen for HG (Figs. 2F, 3F), none of these genes had a significantly altered expression when monocultures of GECs without podocytes were treated with MGO (Fig. 4G). For some of the top up- and downregulated genes in GECs exposed to MGO, we could not detect specific signal by qPCR, e.g., HLF, TCHH, RNF43, which is presumably attributable to their very low expression levels, as observed in the RNA-seq data. The ability of MGO to upregulate ID3 and downregulate FN1 in GECs co-cultured with podocytes was further demonstrated on the protein level (Fig. S4A,B). In line with the qPCR data, no significant changes in the levels of ID3 and FN1 proteins were observed in GEC monocultures exposed to MGO (Fig. S4C). These findings once again emphasise the importance of GEC-podocyte crosstalk for the response to metabolic stimuli linked to DN.

### Validation of the changes in the expression selected genes regulated by HG and MGO in an animal model of DN

In order to determine if changes observed in the co-culture model that we established also occur under DN conditions in vivo, we used leptin-deficient black and tan brachyury (BTBR *ob/ob*) mice. Previous work has shown that BTBR *ob/ob* mice rapidly develop insulin resistance and pronounced hyperglycaemia^29–38^, making them a useful model of type 2 diabetes. Furthermore, a number of studies by us and others have demonstrated that BTBR *ob/ob* mice present multiple hallmarks of diabetic nephropathy, which has made them a widely used model of this disease. In particular, these animals develop progressive albuminuria^29,33,34,36,39,40^, as well as renal lesions with mesangial matrix expansion^30,32,33,41,42^ and glomerular basement membrane thickening^33,41^. Podocyte number and density in *BTBR ob/ob* mice are reduced^32–35,41^ and global retraction and effacement of podocytes is observed^35,41^. Glomerular endothelial cells from these mice lose most of their fenestrae and developed a swollen, vacuolised appearance^41^.

**Figure 5:**
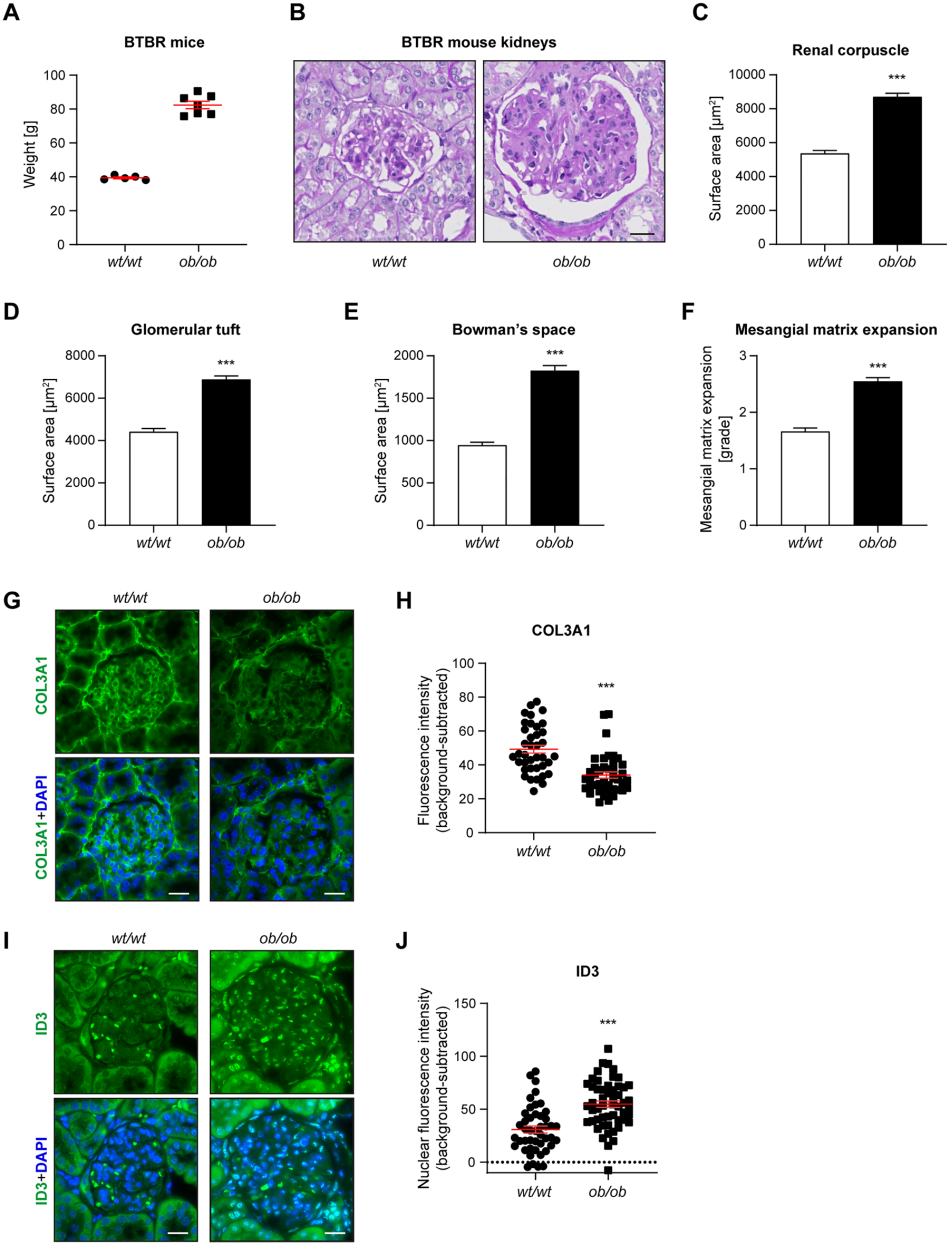
Validation of the changed levels of selected HG- or MGO-regulated genes in the BTBR *ob/ob* mouse model of DN. (**A**) Body weight of the BTBR *wt/wt* (control) and BTRB *ob/ob* female mice that were used for immunofluorescence stainings at ~ 24 weeks of age. (**B**) Representative images of renal corpuscles from kidneys of BTBR *ob/ob* and control (BTBR *wt/wt*) mice, stained using the periodic acid-Schiff (PAS) method. (**C–E**) Morphometric quantification of the surface area of the renal corpuscles (**C**), glomerular tufts (**D**) and Bowman’s space (**E**) in BTBR *wt/wt* and BTBR *ob/ob* mice. (**F**) Pathological assessment of the mesangial matrix, graded on a scale from 1 to 4. (**G–J**) Analysis of immunofluorescence stainings for COL3A1 (downregulated by HG according to the RNA-seq results; **G**,**H**) and ID3 (upregulated by MGO according to the RNA-seq results; **I**,**J**). Representative images of immunofluorescence stainings are shown for COL3A1 (**G**) and ID3 (**I**) in diabetic animals (BTBR *ob/ob*) and non-diabetic controls (BTBR *wt/wt*). The graphs show quantification of background-subtracted COL3A1 signal within glomeruli (**H**, 38 *wt/wt* and 42 *ob/ob* glomeruli, from 5 *wt/wt* and 7 *ob/ob* animals, respectively) and of the background-subtracted ID3 signal within glomerular nuclei (**J**, 45 *wt/wt* and 52 *ob/ob* glomeruli, from 5 *wt/wt* and 7*ob/ob* animals, respectively). The COL3A1 staining (**G**) and ID3 staining (**I**) are shown in green, nuclei were stained with DAPI (blue, **G**,**I**). Scale bars, 20 µm.

We dissected kidneys from BTBR *ob/o*b mice and BTBR *wt/wt* littermates, which served as controls, at ~ 24 weeks of age. As expected, the BTBR *ob/ob* mice had developed obesity with around twice the body weight of controls by that time point (Fig. 5A). Furthermore, histological analysis of the kidneys revealed typical hallmarks of DN, including a significant enlargement of the area of the renal corpuscles, glomerular tufts and Bowman’s space, accompanied by marked expansion of the mesangial matrix in BTBR *ob/ob* mice compared to controls (Fig. 5B–F), in line with our previous findings in these animals^41,42^. To corroborate the RNA-seq results for selected candidates, we performed immunostaining for COL3A1, one of the top downregulated genes by HG in podocytes, as well as for ID3, which was significantly upregulated by MGO in GECs. In agreement with our results in cell culture, COL3A1 levels in renal glomeruli of BTBR *ob/ob* mice were markedly reduced compared to controls (Fig. 5G,H). Within glomeruli, the nuclear expression of ID3 was primarily detected in endothelial cells, as revealed by co-staining with the endothelial cell marker CD31 and the podocyte marker nephrin (Fig. S5A, B). Importantly, the levels of ID3 were significantly increased in BTBR *ob/ob* animals relative to controls (Fig. 5I,J), in line with our findings in the co-culture model. These findings confirm that changes in genes expression we detected following exposure of GECs and podocytes to HG and MGO in culture, which presumably reflect early events in DN, are also observed in a well-established animal model of DN, representative of more advanced stages of the disease.

## Discussion

In this study, we established a co-culture system of human GECs and podocytes to investigate the transcriptomic responses of these cell types to HG and MGO as crucial metabolic challenges in DN, as well as the extent to which the crosstalk between these cells affects their responses.

We observed that the changes in gene expression following these stimuli varied greatly, depending not only on the stimulus, but also on the cell type. GECs showed minimal responses to HG after 48 h, whereas after 96 h of HG, the number of DEGs increased substantially. MGO had the strongest effect on the transcriptional profile of GECs, significantly altering the expression of more than twice as many gene as HG at 96 h. The reverse pattern was observed in podocytes, which showed a rather modest transcriptional response to MGO, but upregulated a considerable number of genes already at 48 h of HG, with a further substantial increase at 96 h of HG. These findings indicate that while both GECs and podocytes can respond to metabolic alteration under DN with considerable changes in gene expression, the contribution of individual metabolic factors is very different for these cell types. Another general observation was that the magnitude of the transcriptional responses to HG and MGO was moderate, with relatively few genes showing over twofold up- or downregulation. However, comparison of 48 vs 96 h HG showed that the changes in expression increased in magnitude after longer treatment, suggesting that long-term exposure to diabetic metabolites can have cumulative effects over the course of the disease.

The top genes upregulated by HG in both GECs and podocytes belong to the category of the immediate early response genes. The latter were originally named based on their ability to respond rapidly and transiently to various stimuli, but more recent work has shown that sustained expression of such genes can be linked to different pathological conditions^28^. Our results show that members of the early growth response family of transcription factors, EGR1, EGR2 and/or EGR3, were at the top of the upregulated gene lists in both GECs and in podocytes after exposure to HG. The observed upregulation of EGR1 by HG in GECs and podocytes is in line with previous reports^43,44^, and our data suggest that other EGR family members are similarly altered. EGR1 has been linked to DN pathogenesis by a number of studies, but these have demonstrated roles for EGR1 in other cell types of the kidney, including mesangial cells^45–50^ and tubular epithelial cells^51–53^. By contrast, little is known so far about a potential role of EGR family members in GECs or podocytes, and our data suggest that EGR transcription factors could play a similarly important role in both of these cell types under hyperglycaemic conditions. Another immediate early transcription factor that was prominently upregulated by HG in GECs was NR4A1 (nuclear receptor subfamily 4 group A member 1). The latter has been shown to be activated by HG in a murine DN model, which was associated with renal dysfunction^54^, but the cells types that upregulate NR4A1 in response to the hyperglycaemic stimulus have not been identified. Our results indicate that GECs play an important role in this process.

In GECs, HG led to upregulation of genes involved in inflammatory and immune responses, with a number of cytokines, chemokines and interleukins (e.g. CXCL1, CXCL3, CXCL6, CXCL8, CSF2/3, IL1A/B, IL-6), as well as regulators of TNF and NF-κB and signalling (e.g. NFKBIZ and TNFAIP3) among the top upregulated genes. MGO similarly upregulated IL-24 in GECs, a cytokine that is associated with chronic inflammation. Several of these inflammatory signalling molecules have been implicated in the pathogenesis of DN. For example, increased inflammation and CXCL1 expression were observed in the kidneys of both diabetic mice and DN patients, and inhibition of CXCL1 signalling ameliorated renal damage in a murine DN model^55^. Similarly, CXCL8 was found to be elevated in DN patients and diabetic mice, and treatment with a CXCL8-specific antagonist improved histological and functional parameters related to DN development in murine diabetic models^56,57^. In general, inflammation plays a crucial role in promoting the development of DN^58^ and our findings indicate that changes directly induced by HG in GECs could contribute to the establishment of an inflammatory environment early on during the disease, affecting its progression.

Given the increased expression of pro-inflammatory cytokines and chemokines in GECs upon exposure to HG and MGO, we speculate that continuous exposure of GECs to these metabolites in the kidney likely contributes to the development of a chronic inflammatory environment. Chronic inflammation, in turn, can induce fibrosis, for example through the activation of fibroblasts through growth factors, chemokines and cytokines released by inflammatory cells^59^. IL-1β and IL-6—both induced in GECs after exposure to HG—are good examples of such cytokines^60,61^. From this perspective, it is also notable that the NLRP3 inflammasome, a central component of the chronic inflammatory response, is emerging as a mediator of diabetic tubular damage and renal fibrosis^62^, and is positively regulated through CXCL1/2^63^, cytokines that are induced by HG in GECs. CXCL6 was also found to be overexpressed in DN patients and was upregulated by HG in renal fibroblasts, which was associated with renal fibrosis^64^. In view of these findings, it is interesting to speculate that chronic metabolite-induced changes in the expression of inflammatory signalling molecules as a consequence of diabetes might provide a link between the early deterioration of kidney function in the glomerulus and the chronic inflammation-dependent tubular damage and fibrosis that ultimately leads to end-stage renal disease.

Among the genes that were downregulated by HG in GECs, the top overrepresented biological process was the response to hypoxia. Interestingly, it has been reported that ischemic preconditioning protects GEC function against oxygen and glucose deprivation (as a model of ischemia–reperfusion injury) under normoglycaemia, but fails to do so under hyperglycaemia^65^. These observations could be attributable to the dampened response to hypoxia that our gene expression analyses revealed in GECs under HG, as hypoxic signalling plays a key role under ischemic conditions.

The finding that genes involved in ECM organisation were downregulated by HG in podocytes and by MGO in endothelial cells was unexpected, since thickening of the glomerular basement membrane, accompanied by increased production of several ECM components, is a histopathological hallmark of DN^66^. While the explanation of these results is currently unclear, they could indicate that HG and MGO are not directly responsible for the induction of most ECM components that contribute to increased basement membrane deposition. Indeed, thickening of the glomerular basement membrane is observed at least 1–2 years after the clinical onset of diabetes and it is perfectly possible that other alterations, secondary to HG or MGO (e.g., activation of inflammatory signals^67,68^) could be the main driving force behind enhanced ECM production and remodelling in DN. On the other hand, an initial reduction in ECM components upon challenge with HG and/or MGO could conceivably activate compensatory mechanisms that eventually overshoot, resulting in abnormally increased ECM deposition over time. At the same time, while the bulk of the ECM increase may be contributed by other factors, HG could still play a role in upregulating some ECM components—for example, even if ECM-related functional groups were not overrepresented in the upregulated genes in podocytes after HG, one of the top upregulated genes in podocytes after HG (*LAMA4*) belongs to the family of laminins, which is elevated in DN^67^.

Previous work using a diabetic rat model induced by administration of streptozotocin and high fat diet indicated that the expression of COL3A1, which encodes type III collagen, is increased in the kidneys, but decreased in the heart of the hyperglycaemic animals^69^. In addition, COL3A1 was among the genes elevated in patients with diabetic nephropathy within tubulointerstitial tissue^70^. Our results, on the other hand demonstrate that COL3A1 was among the ECM genes that were downregulated by HG in podocytes and our immunostaining analysis confirmed this finding within the glomeruli of BTBR *ob/ob* mice. This suggests that the expression of COL3A1 is differentially affected by hyperglycaemic conditions in different compartments and within different cell types of the kidney.

The ID (inhibitor of DNA binding / inhibitor of differentiation) family includes several helix-loop-helix proteins, which control the activity of diverse transcription factors and thereby play key roles in the development of various tissues and organs, as well as in disorders linked to them^71^. Among the cell types with highest expression of ID proteins, in particular of the best-studied members ID1 and ID3, are endothelial cells, where ID1 and ID3 have been shown to play a central role in angiogenesis both during normal development and in different pathophysiological conditions^72–74^. Our gene expression analyses demonstrate that ID1 and ID3 are among the prominently upregulated proteins in GECs exposed to MGO. This finding was further confirmed for ID3 in the BTBR *ob/ob* model. Studies using genetic deletion of ID1 and ID3 in animal models have provided evidence that they play functional roles in protecting the kidney vasculature against different pathological challenges, including ischemia–reperfusion injury^75^, hyperlipidaemia^76^ and irradiation^77^. Both ID1 and ID3 were upregulated by high glucose in cultured human pancreatic islets or insulinoma cells^78^. Furthermore, the expression of ID1 has been shown to increase markedly in a streptozotocin model of diabetes and ID1 knockout in this model exhibited accelerated pathological alterations in the kidneys^79^. Less is known at present about the specific role of ID3 in the context of diabetic nephropathy, but our findings suggest that it may also play an important role in its pathogenesis. It is conceivable, for instance, that both ID1 and ID3 are upregulated as a protective mechanism against diabetic insults, which may delay but eventually fail to prevent the onset of nephropathy. At same time, it is also feasible that sustained upregulation of ID proteins may contribute to the pathogenesis of DN, e.g. by impacting cell differentiation in ways that compromise normal GEC function^80,81^ or by inducing inflammation^82^.

A key finding of our study is that the crosstalk between GECs and podocytes is crucial in controlling their responses to HG and MGO. Strikingly, none of the top genes that we validated as up- or downregulated in co-cultures of GECs and podocytes were differentially expressed in monocultures of the respective cells. A considerable body of literature has shown that GECs and podocytes extensively communicate, influencing each other’s properties and functions. This crosstalk can be mediated by a variety of secreted factors^14,15^. For example, podocytes produce large amounts of VEGF-A, which can bind to VEGF (co)receptors on the surface of GECs. VEGF-A expression is increased in diabetic models and podocyte-specific deletion of VEGF-A caused GEC damage, accelerating the progression of glomerular injury and dysfunction^83^. Similarly, GEC-specific loss of BMP and activin membrane bound inhibitor (BAMBI), a negative modulator of TGF-β signalling, resulted in podocyte loss and faster DN progression in a murine model of streptozotocin-induced diabetes^84^. Further work has demonstrated that podocytes can induce mitochondrial dysfunction and oxidative stress in GECs via TGFβ-dependent secretion of endothelin-1^85,86^, which was also upregulated in podocytes exposed to HG in our experiments (Supplementary Table S1). This, in turn, has been associated with podocyte depletion in a mouse model of DN^87^, as well as in a co-culture system of GECs and podocytes exposed to HG^88^. The interactions between GECs and podocytes further modulate the activation of downstream inflammatory responses, as shown by the finding that the TNFα-mediated recruitment of neutrophils by GECs was significantly inhibited in co-cultures with podocytes, compared to GEC monocultures. This effect was dependent on the secretion of IL-6 that was predominantly released by podocytes^89^. The crosstalk between GECs and podocytes has also been shown to play a critical role for the composition of the ECM produced by these cells. Co-culture of the two cells types resulted in the production of ECM, which had a different composition compared to that produced by GEC or podocyte monocultures and more closely resembled tissue-derived glomerular ECM^90^. In the co-culture system that we employed, the pore size of the separating membrane (0.4 µm) allows diffusion not only of soluble factors, but also of small extracellular vesicles such as exosomes that can also carry signals important for the GEC-podocytes crosstalk. Indeed, it has been shown that HG-treated GECs secrete more exosomes than normoglycaemic control cells. These exosomes can promote epithelial-mesenchymal transition (EMT) in podocytes through a mechanism that was proposed to depend on TGF-β1 mRNA, which was enriched in the exosomes, and canonical Wnt/β-catenin signalling^91^. Interestingly, the gene for E-cadherin (CDH1), whose repression is a hallmark of EMT, was among the top downregulated genes in podocytes exposed to HG in co-cultures with GECs (Fig. 3B), potentially consistent with an induction of an EMT-like phenotype in these conditions.

Our experimental system has certain limitations. For instance, while the glomerular endothelial cells and podocytes were cultured together, they were separated by a greater distance than in the glomerulus, which could impact the local concentration of soluble factors. Furthermore, the podocytes in our case were grown on a planar membrane, but the topography and curvature of the substrate on which they are cultured can affect their differentiation and other properties^92^. In addition, the BTBR *ob/ob* animals used in this study were female. Most previous work has shown a broadly similar phenotype in male and female BTBR *ob/ob* mice, e.g. in terms of body weight, insulin and glucose levels^29,31,38^. Furthermore, male and female mice show no significant differences in albuminuria and histological parameters of renal lesions including glomerular mesangial matrix accumulation or reduction in podocyte number and density^29,33^. Nevertheless, some differences have also been reported, including a greater elevation of triglycerides and a more pronounced decrease in intraepidermal nerve fibre density in male vs female BTBR *ob/ob* mice^93^. Thus, it remains feasible that some of the gene expression alterations we detected may differentially affect male and female BTBR *ob/ob* mice, a possibility that can be explored in future work. Ultimately, validation of our findings in diabetic nephropathy patients at different stages of the disease can provide the most robust evidence for their potential clinical applicability.

The above-mentioned limitations notwithstanding, our system allowed us to study the impact of distinct metabolic parameters characteristic of DN on GECs and podocytes, as well as the effect that the crosstalk between these cells has on their response to such factors. Our finding that GECs and podocytes prominently affect each other’s responses to HG and MGO indicates that previous studies using monocultures of individual cell types may have limitations in revealing the full range of responses to such stimuli in the glomerular environment. Recent work using single-cell sequencing in DN models^18,19^ and patients^21^ have allowed detailed characterisation of the transcriptomic changes induced in distinct renal cell types within their natural environment. However, such studies have assessed kidneys in which DN is already established and cannot typically be reverted. By contrast, our study provides analysis of the early changes occurring in response to the key diabetes-related metabolic challenges HG and MGO in GECs and podocytes that are able to interact with each other. The information gained in this manner can potentially serve as a basis for identifying mechanisms leading to the initial development of DN, which can be counteracted to prevent the onset of the disease.

## Materials and methods

### Cell culture

Conditionally immortalised GECs (obtained from the University of Bristol) were cultivated using endothelial cell growth medium (EBM-2, Lonza) supplemented with 5% FCS and growth factors (hFGF, R3-IGF, hEGF), GA-1000, ascorbic acid and hydrocortisone (Lonza). Conditionally immortalised podocytes (obtained from the University of Bristol) were cultivated in 10 cm dishes in RPMI-1640 medium (Thermo Fisher Scientific) supplemented with 10% FBS (Gibco), 1 g/L glucose (Roth), 2 g/L sodium hydrogen carbonate (Roth), insulin-transferrin-selenium (Gibco) and 200 U/mL penicillin/streptomycin (Gibco). To maintain proliferation, cultures were kept at 33 °C, 5% CO_2_ and 95% relative humidity. After reaching around 80% confluence, the cells were passaged using 0.25% Trypsin/EDTA (Gibco), and experiments were performed on cells in passages 2–4 after thawing. Medium was changed every 2–3 days.

### Co-culture of glomerular endothelial cells and podocytes

Podocytes were seeded at a density of 50,000/well on top of 24 mm polycarbonate membrane inserts with 0.4 µm pores in 6-well Transwell chambers (Corning, 3412) and left for four days under proliferation conditions (33 °C). To induce differentiation and inactivate the SV40 large T antigen, cultures were transferred to 37 °C. While podocytes were differentiating, GECs with a density of 60,000/well were seeded separately on the bottom surface of 6-well chambers for four days under proliferation conditions, before switching to differentiation conditions as above. The differentiation of podocytes lasted for 14 days, whereas that of GECs lasted four days, as previously established^25–27^. The seeding of the cells was timed in such a manner that the end of the differentiation periods for the GECs and podocytes would coincide (see Fig. 1A). The transwell inserts containing the differentiated podocytes were then combined with the 6-well chambers containing the GECs and exposed for 48 h or 96 h to hyperglycemic conditions (25 mM glucose, HG) or for 96 h to 200 µM methylglyoxal (MGO, Sigma M0252) in RPMI-1640 medium. The control group for HG contained 5.5 mM glucose and 19.5 mM mannitol as osmotic control. Water served as control for the MGO treatment. During HG and MGO stimulation, the medium was refreshed once per day.

### Viability assay

GECs and podocytes were plated in 96-well plates at a density of 3000 cells/well. After proliferation and differentiation phases, as described above, cell viability/number was determined using CyQUANT NF Cell Proliferation Assay (Thermo Fisher Scientific) following the manufacturer’s instructions. Briefly, the culture medium was gently removed and 50 µL of of CyQUANT dye binding solution were dispensed into each well. The microplate was incubated for 45 min with the dye and fluorescence intensity was measured with excitation at 485 nm and emission detection at 530 nm using a Microplate Reader (Infinite M200, Tecan).

### RNA isolation and RNA sequencing

Total RNA was prepared using an RNeasy mini kit (Qiagen) according to the manufacturer’s instructions and eluted in 30 µL nuclease-free water. Prior to isolation, co-cultures were rinsed with phosphate buffered saline (PBS, Gibco). Then, RNA was isolated separately from the podocytes cultivated on the inserts and the GECs cultivated at the bottom of the transwell chambers. The quality of the RNA was assessed by Nano-Drop measurements and electrophoresis on a 2100 Bioanalyzer (Agilent). From each group and each cell type, four separate RNA samples were selected for RNA-seq based on RNA quality (all samples had RIN [RNA-integrity number] of at least 9.7 out of 10). RNA sequencing was performed by BGI Genomics using the BGISEQ 500 and DNBSEQ platforms. For monoculture experiments, RNA isolation was performed in the same manner.

### RNA-seq data analysis

Most of the RNA-seq data analysis was carried out with R and Bioconductor using the NGS analysis package systemPipeR^94^. Quality control of raw sequencing reads was performed using FastQC (Babraham Bioinformatics). Low-quality reads were removed using trim_galore (version 0.6.4). The resulting reads were aligned to human genome version GRCh38.p13 from GeneCode and counted using kallisto version 0.46.1^95^. The count data were transformed to log2-counts per million (logCPM) using the voom-function from the limma package^96^. Differential expression analysis was performed using the limma package in R. A false positive rate of α = 0.05 with FDR correction was taken as the cutoff for significance. Heatmaps were generated using the Morpheus visualisation and analysis tool (https://software.broadinstitute.org/morpheus/). Volcano plots were generated using VolcaNoseR (https://huygens.science.uva.nl/VolcaNoseR/;^97^). All hits with a logFC > 1 and FDR-corrected p-value < 0.05 were marked in red (strongly upregulated genes) and all hits with a logFC < − 1 and FDR-corrected p-value < 0.05 were marked in blue (strongly downregulated genes). Venn diagrams were created using Venn Diagram Plotter Version 1.5 (https://github.com/PNNL-Comp-Mass-Spec/Venn-Diagram-Plotter/releases/tag/v1.6.7458).

Functional annotation analysis was performed using the database for annotation, visualisation and integrated discovery (DAVID, Version 1.7: https://david.ncifcrf.gov/tools.jsp;^98^). Gene ontology biological processes terms were plotted. To explore potential interactions between genes belonging to overrepresented functional groups, we performed STRING analysis (v. 11.5, https://string-db.org/;^99^). The interaction score threshold was set to 0.7 (high confidence). The following colour codes were used to denote the types of interactions (connecting lines) in the STRING networks: cyan, known interactions from curated databases; magenta, experimentally determined interactions; green, interactions predicted on the basis of gene neighbourhood; red, interactions predicted on the basis of gene fusions; blue, interactions predicted on the basis of gene co-occurrence; light green, interactions predicted on the basis of text mining; black, interactions predicted on the basis of co-expression; light purple, interactions predicted on the basis of protein homology.

### Reverse transcription quantitative PCR (qPCR)

DNA digestion was performed using RNase free DNase (Thermo Fisher Scientific). A total of 1 µg RNA was reversed-transcribed into cDNA using RevertAid H minus reverse transcriptase (Thermo Fisher Scientific) according to the manufacturer’s instructions, and eluted in 100 µL DEPC water. Quantitative PCR was performed on a QuantStudio3 qPCR-System (Applied Biosystems) using the GoTaq qPCR kit (Promega) and the following thermal cycling profile: 2 min at 95 °C, followed by 15 s at 95 °C and 1 min at 60 °C (repeat for 40 cycles). For all samples, gene expression was normalised to RPLP0 as a house keeping gene, and results were calculated relative to the control group. Primers were designed using NCBI Primer-BLAST and product-specificity was evaluated by gel electrophoresis and melt curve analysis. The sequences of the primers used for qPCR are listed in Supplementary Table S3.

### Gel electrophoresis and Western blotting

Cells were lysed directly in the transwell insert or the 6-well plate using SDS sample lysis buffer (4% SDS, 125 mM Tris–HCl pH 6.8, 20%, glycerol in double-distilled water) and sonicated using a Sonopuls GM 2070 tip sonicator (Bandelin) to fragment DNA and decrease viscosity. Protein concentration was determined using the Pierce BCA Protein Assay Kit (Thermo Fisher Scientific). Equal amounts of total protein (30–50 μg per lane) were mixed with 6 × Laemmli sample buffer, heated at 95 °C for 5 min and loaded on a sodium dodecyl sulfate polyacrylamide gel. After electrophoretic separation, proteins were electrotransferred onto an Amersham Protran 0.2 µm nitrocellulose blotting membrane (Cytiva) at 4 °C overnight using wet transfer. After blocking with 5% skimmed milk in PBST (136.9 mM NaCl, 2.7 mM KCl, 8 mM Na_2_HPO_4_ × 2H_2_O, 1.5 mM KH_2_PO_4_, 0.1% Tween 20, pH 7.4) for 1 h, proteins of interest were detected using overnight incubation at 4 °C with the following primary antibodies: ID3 rabbit monoclonal antibody (Biocheck, clone 17-3, BCH-4/#17-3, final concentration 1.3 µg/mL); fibronectin (FN1) rabbit polyclonal antibody (Abcam, ab23750, final concentration 1 µg/mL); β-actin mouse monoclonal antibody (Sigma, clone AC-15, A5441, ascites, final dilution 1:20,000–1:50,000), α-tubulin mouse monoclonal antibody (Abcam, clone B-5-1-2, ab11304, final concentration 0.05 µg/mL). After washing 3 times with TBST (50 mM Tris, 146 mM NaCl, 0.1% Tween 20, pH7.6), the membranes were incubated for 2 h with horseradish peroxidase (HRP)-conjugated secondary antibodies: HRP goat anti-rabbit IgG, Dako, P0448, final concentration 0.17 µg/mL), HRP goat anti-mouse IgG, Dako, P0447, final concentration 0.5 µg/mL). After additional three washes, the signal was developed using chemiluminescence substrates (Thermo Fisher Scientific and Peqlab) and imaged on a Fusion Solo S Imager (Vilber Lourmat). Protein levels were quantified densitometrically using ImageJ/Fiji^100^ and normalised to the loading control (actin or tubulin).

### Animal experiments

The experiments using the BTBR *ob/ob* DN model were approved by the local animal welfare officer of the Medical Faculty Mannheim, University of Heidelberg (AZ I-20/19), conducted according to the applicable guidelines and regulations and reported in accordance with ARRIVE guidelines. BTBR mice heterozygous for leptin deficiency (BTBR *wt/ob*) were purchased from Jackson Laboratories (strain #: 004824) and bred in the in-house specific pathogen-free animal facility. Diabetic BTBR *ob/ob* mice and or non-leptin deficient BTBR *wt/wt* mice (control group) were fed regular chow ad libitum and kept at 22 °C in a light–dark cycle of about 12 h. At an age of 23–25 weeks, body weight was measured, and then mice were sacrificed by cervical dislocation. The right kidney of each mouse was fixed with 4% paraformaldehyde and embedded in paraffin. Kidneys from seven female BTBR *ob/ob* and five female BTBR *wt/wt* mice were used for the immunostainings described below.

### PAS staining and histological analysis

Paraffin-embedded kidneys were cut in 3 µm sections. Xylol was used for deparaffinisation and an ethanol gradient for dehydration. Sections were stained with periodic acid Schiff (PAS) reagent (P7875, Sigma-Aldrich) and counterstained with haematoxylin (1.09249, Sigma-Adrich and H02-1000, Dr. K. Hollborn & Söhne, 1:1 mixture). Stained slides were digitalised for morphometric analysis using an Axio Scan.Z1 slide scanner (40 × magnification) and ZEN 3.1 microscopy software (blue edition) (both Carl Zeiss Microscopy). To quantify histological changes of BTBR *ob/ob* compared to BTBR *wt/wt* mice, renal corpuscle area and glomerular tuft area of 20 randomly selected glomeruli per animal were measured using QuPath version 0.4.4^101^. Bowman’s space area was calculated through subtraction of the glomerular tuft area from the renal corpuscle area. Mesangial matrix expansion was assessed by a pathologist with expertise in nephropathology (T.A.) in a blinded manner. To this end, the amount of mesangial matrix compared to the glomerular tuft area was analysed in 20 glomeruli of each animal, which were graded on a scale of 1 to 4, defined as follows: 1, 0–10%; 2, 10–50%; 3, > 50%, 4, glomerular sclerosis.

### Immunofluorescence staining

Paraffin-embedded kidneys were cut in 3–4 µm sections and deparaffinised as described above. For immunostaining, antigen retrieval was performed using Tris/EDTA-buffered target retrieval solution pH 9 (Dako) for 20 min at 100 °C. For COL3A1, antigen retrieval was performed with Proteinase K solution (Dako) for 5 min at room temperature. Rabbit polyclonal anti-collagen-III (COL3A1) antibody (ab7778, abcam; 1:100), rabbit polyclonal anti-ID3 antibody (ab41834, abcam; 1:200), rat monoclonal anti-CD31 (clone SZ31, DIA-310, Dianova; 1:20) and goat polyclonal anti-nephrin antibody (AF3159, R&D Systems, 1:40) were used as primary antibodies. For single stainings of ID3 and collagen III, goat anti-rabbit F(ab')2, Alexa Fluor 488 (A11070, Thermo Fisher Scientific; 1:1000) was used as a secondary antibody. For co-staining of ID3 and CD31, goat anti-rabbit F(ab')2, Alexa Fluor 488 (A11070, Thermo Fisher Scientific; 1:1000) and goat anti-rat IgG Alexa Fluor 546 (A11081, Thermo Fisher Scientific; 1:1000) were used as secondary antibodies. For co-staining of ID3 and nephrin, donkey anti-rabbit 488 (Jackson 711-545-152, Jackson ImmunoResearch; 1:400) and donkey anti-goat 555 (Jackson 705-165-147, Jackson ImmunoResearch; 1:400) were used as secondary antibodies. Slides were blocked using 10% goat serum, 1% BSA in PBS for 1 h at room temperature for single ID3 and collagen III stainings, and for co-staining of ID3 and CD31. For co-staining of ID3 and nephrin, blocking was done with 10% donkey serum. After blocking, slides were incubated with primary antibodies at 4 °C overnight. Following rinsing 3 × 5 min with PBS, sections were then incubated with secondary antibodies for 1 h at room temperature. For co-stainings, both primary antibodies and secondary antibodies were incubated simultaneously. Subsequently, the slides were rinsed again as above, stained with DAPI (1 µg/mL) for 7 min, rinsed 3 × 5 min in PBS and mounted with Fluoromount G (Thermo Fisher Scientific). For each animal, a section for which the primary antibodies were omitted was used as a negative control for background staining. The single stainings were imaged using an AxioImager D1 microscope and ZEN software (Carl Zeiss Microscopy). The co-stainings were imaged on an LSM 800 confocal microscope (Carl Zeiss Microscopy).

### Image quantification

Immunofluorescence images were analysed using ImageJ/Fiji^100^. A minimum of five randomly selected glomeruli were imaged from each animal. For quantification of the ID3 staining, the signal was only measured in the nuclear regions within the glomerulus, as defined by DAPI staining. Specifically, the following steps were followed: (1) the glomerulus was selected and all signal outside of the glomerulus was cleared; (2) within the glomerulus, the nuclear area was selected by masking the DAPI positive region; (3) the ID3 signal was measured within the nuclear area only; (4) the total ID3 signal was divided by the total nuclear area; (5) the same procedure (steps 1–4) was performed for negative control glomeruli (stained with secondary antibody only) from the same animal; (6) the signal of the negative control glomeruli was taken to represent background and was subtracted from the signal measured in the ID3-stained glomeruli of the same animal, as a sample-specific threshold for fluorescence signal intensity. A similar procedure, but without selection of the nuclear area, was followed for the quantification of the COL3A1 signal, namely: (1) the glomerulus was selected and all signal outside of the glomerulus was cleared; (2) the COL3A1 signal was measured within the glomerular area only; (3) the total COL3A1 signal was divided by the total glomerular area; 4) the same procedure (steps 1–3) was performed for negative control glomeruli (stained with secondary antibody only) from the same animal; (5) the signal of the negative control glomeruli was taken to represent background and was subtracted from the signal measured in the COL3A1-stained glomeruli of the same animal, as sample-specific threshold. The signal for an individual glomerulus was used as a data point in the quantifications. The experimenters were not blinded for the quantification.

### Statistical analysis

All data are expressed as mean ± SEM, the values of individual replicates in bar graphs are depicted as grey triangles. For pairwise comparisons, statistical analysis was performed using unpaired, two-tailed Student’s t-test. A p-value < 0.05 was set as a threshold for statistical significance: *p < 0.05,**p < 0.01, ***p < 0.001; ns p ≥ 0.05 (not significant).

### Supplementary Information

Supplementary Information 1.

Supplementary Table S1.

Supplementary Table S2.

## Data Availability

The RNA-seq data have been deposited at the Gene Expression Omnibus (GEO), with accession number GSE220229. The other datasets generated during the current study are available from the corresponding authors on reasonable request.

## Abbreviations

BTBR: Black and tan brachyury
DEG(s): Differentially expressed gene(s)
DN: Diabetic nephropathy
EMT: Epithelial-mesenchymal transition
GEC(s): Glomerular endothelial cell(s)
HG: Hyperglycaemia
MGO: Methylglyoxal
