# Supplementary material for: The crosstalk between glomerular endothelial cells and podocytes controls their responses to metabolic stimuli in diabetic nephropathy
Source: Sci Rep. 2023 Oct 20;13:17985. doi: 10.1038/s41598-023-45139-7 (PMC10589299; doi:10.1038/s41598-023-45139-7)
Supplement: Supplementary file 1 — Supplementary Information 1. [file 41598_2023_45139_MOESM1_ESM.pdf]

SUPPLEMENTARY INFORMATION

The crosstalk between glomerular endothelial cells and podocytes controls their responses to metabolic stimuli in diabetic nephropathy

Michael Albrecht[1,2], Carsten Sticht[3,4], Tabea Wagner[1,2], Steffen A. Hettler[5], Carolina De La Torre[3,4], Jiedong Qiu[5], Norbert Gretz[3], Thomas Albrecht[6], Benito Yard[5], Jonathan P. Sleeman[1,2,7,*], Boyan K. Garvalov[1,2,*]

[1]European Center for Angioscience (ECAS), Medical Faculty Mannheim of the University of Heidelberg, Ludolf-Krehl-Strasse 13–17, 68167 Mannheim, Germany

[2]Mannheim Institute for Innate Immunoscience (MI3), Medical Faculty Mannheim of the University of Heidelberg, Ludolf-Krehl-Strasse 13–17, 68167 Mannheim, Germany

[3]Center of Medical Research, Bioinformatics and Statistics, Medical Faculty Mannheim of the University of Heidelberg, Theodor-Kutzer-Ufer 1-3, 68167 Mannheim, Germany.

[4]NGS Core Facility, Medical Faculty Mannheim of the University of Heidelberg, Theodor-Kutzer-Ufer 1-3, 68167 Mannheim, Germany

[5]Department of Nephrology, Hypertensiology, Endocrinology, Diabetology, Rheumatology and Pneumology, Fifth Department of Medicine, Medical Faculty Mannheim of the University of Heidelberg, Mannheim, Germany.

[6]Institute of Pathology, University Hospital Heidelberg, Im Neuenheimer Feld 224, Heidelberg, Germany.

[7]Institute of Biological and Chemical Systems – Biological Information Processing (IBCS-BIP), Karlsruhe Institute of Technology Campus North, Building 319, Hermann-von-Helmholtz-Platz 1, 76344, Eggenstein-Leopoldshafen, Germany

*These authors contributed equally: Jonathan P. Sleeman and Boyan K. Garvalov

SUPPLEMENTARY FIGURES

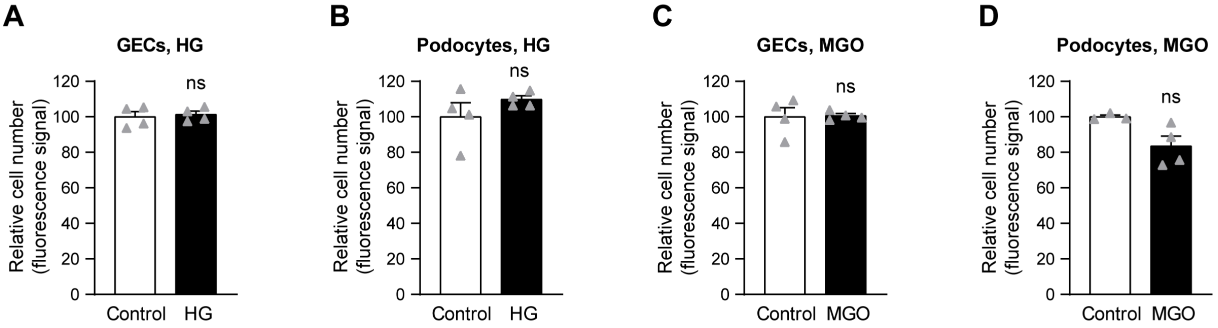

Figure S1. HG and MGO do not significantly affect the number of GEC or podocytes. (**A-D**) GECs (**A**, **C**) and podocytes (**B**, **D**) were differentiated and treated with 25 mM glucose (HG, addition of 19.5 mM glucose to RPMI-1640 medium containing 5.5 mM glucose, **A**, **B**) or with 200 µM MGO (**C**, **D**) for 96 hr as described (see Fig. 1A). 5.5 mM glucose + 19.5 mM mannitol (osmotic control) and water served as treatment controls, respectively. Cell viability / cell numbers were assessed using a CyQUANT Proliferation Assay and the fluorescence signal was normalised relative to the control condition (n=3-4).

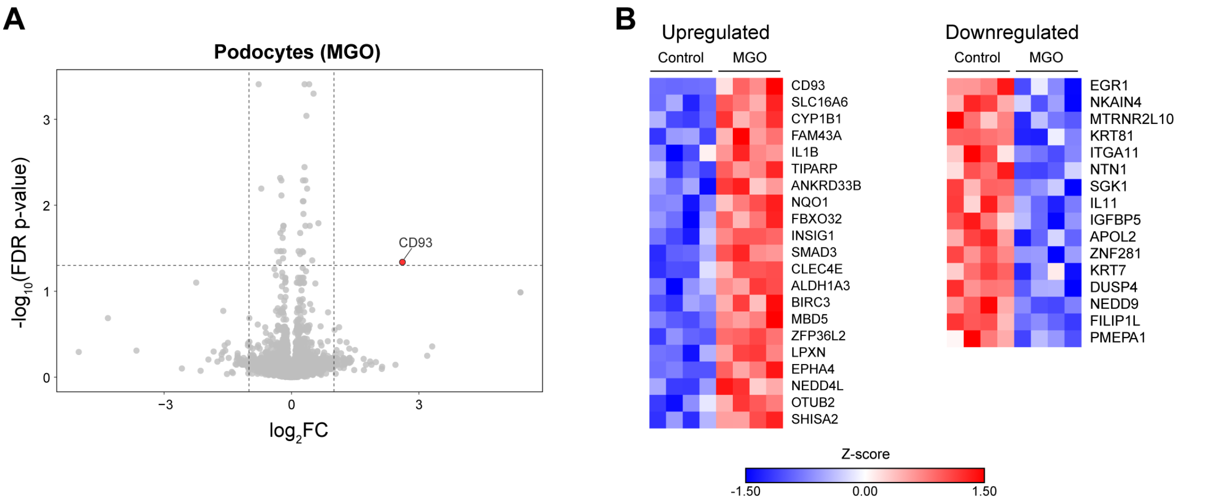

Figure S2. MGO has limited effects on podocytes. (**A**) Volcano plot of gene expression changes in podocytes exposed to 96 hr of MGO compared to control cells. Genes with $log_2FC > 1$ and FDR-corrected p-values <0.05 were marked in red (strongly upregulated genes). (**B**) Heat maps of all up- and downregulated genes in GECs after 96 hr of MGO.

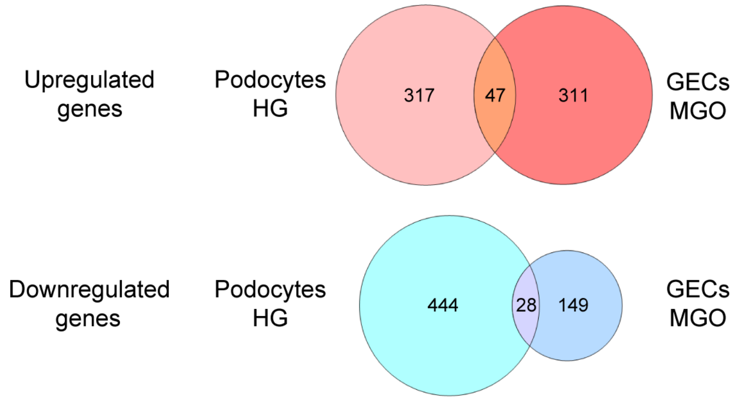

Figure S3. Limited overlap between the genes regulated by HG in podocytes and by MGO in GECs. Venn diagrams showing the overlaps between DEGs in podocytes exposed to HG and GECs exposed to MGO for 96 hr. Upregulated genes sets are represented by red hues, downregulated gene sets – by blue hues.

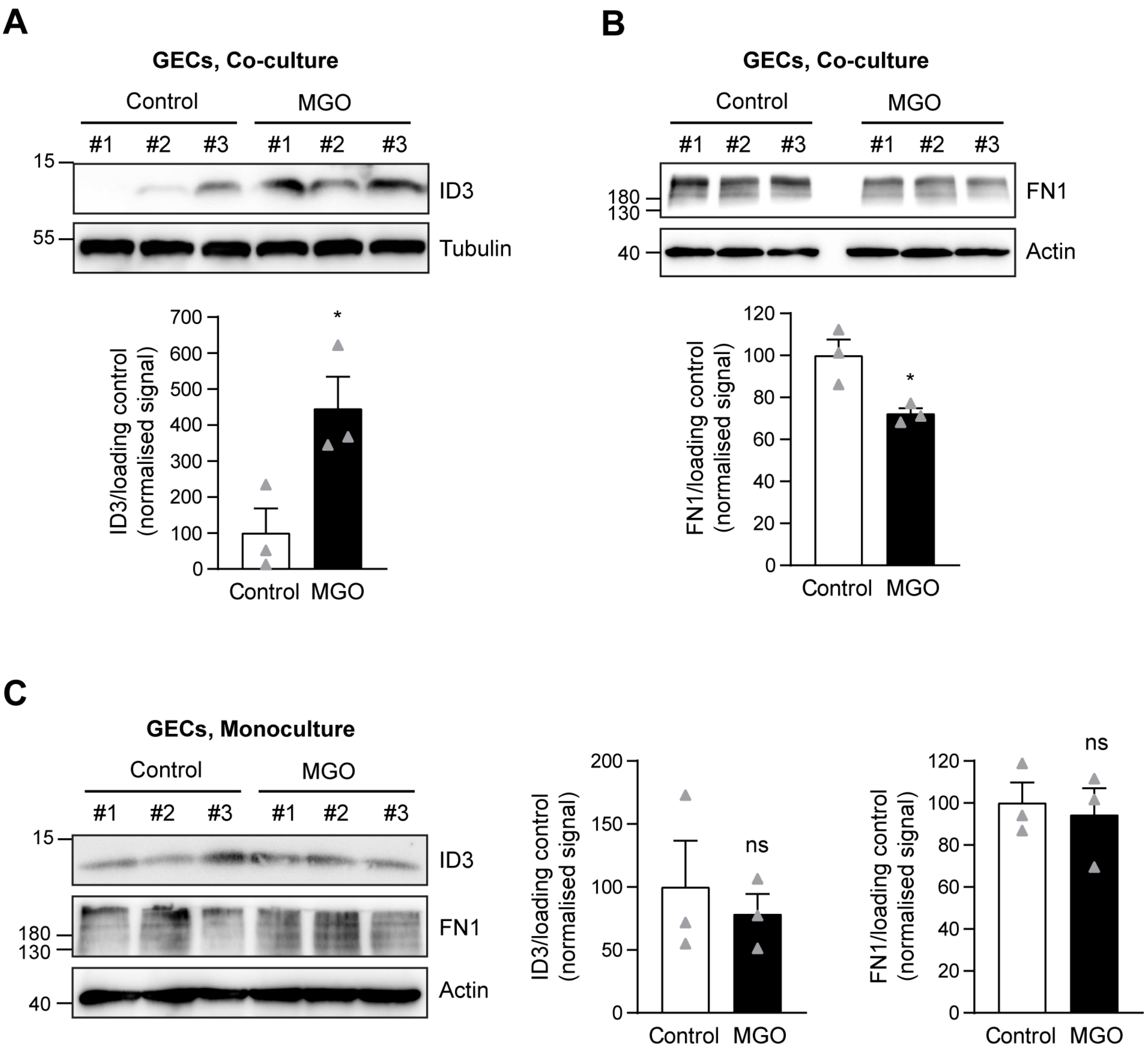

Figure S4. MGO treatment of GECs increases ID3 and decreases FN1 protein levels in co-cultures with podocytes, but not in monocultures. (**A**, **B**) GECs were co-cultured with podocytes as described (see Fig. 1A) and exposed to 200 µM MGO for 96 hr; water served as treatment control. GECs were lysed and analysed by Western blot to determine the protein levels of ID3 (**A**) and FN1 (**B**). Actin and tubulin served as loading controls. The graphs under the Western blots present densitometric quantification of protein levels normalised to loading controls for the separate replicates (n=3). (**C**) GECs were cultivated in monoculture, exposed to 200 µM MGO for 96 hr and the protein levels of ID3 and FN1 were determined by Western blot. The graphs next to the Western blots present densitometric quantification of protein levels normalised to loading controls for the separate replicates (n=3).

A

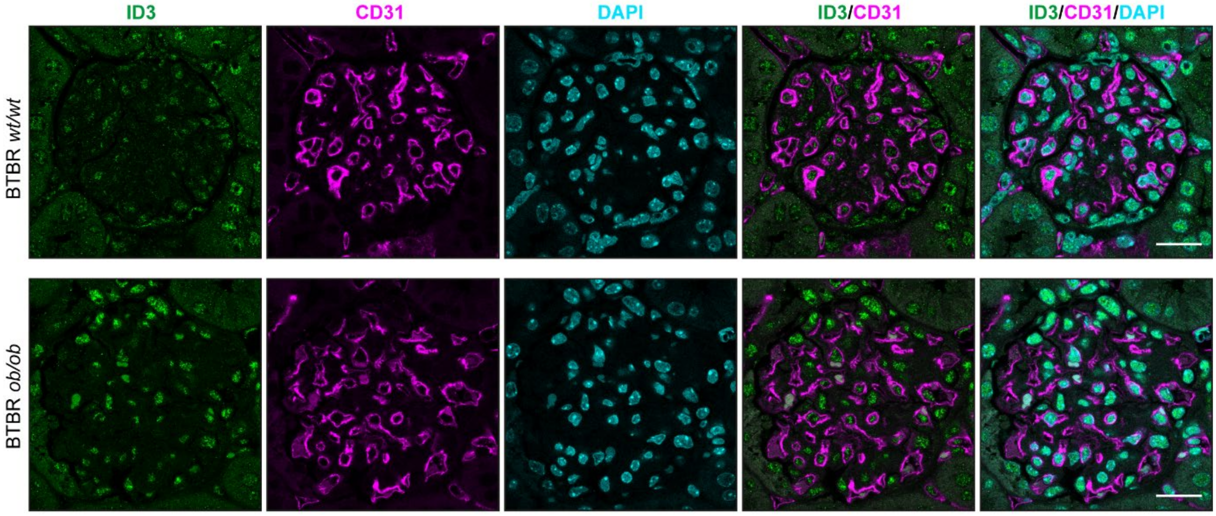

B

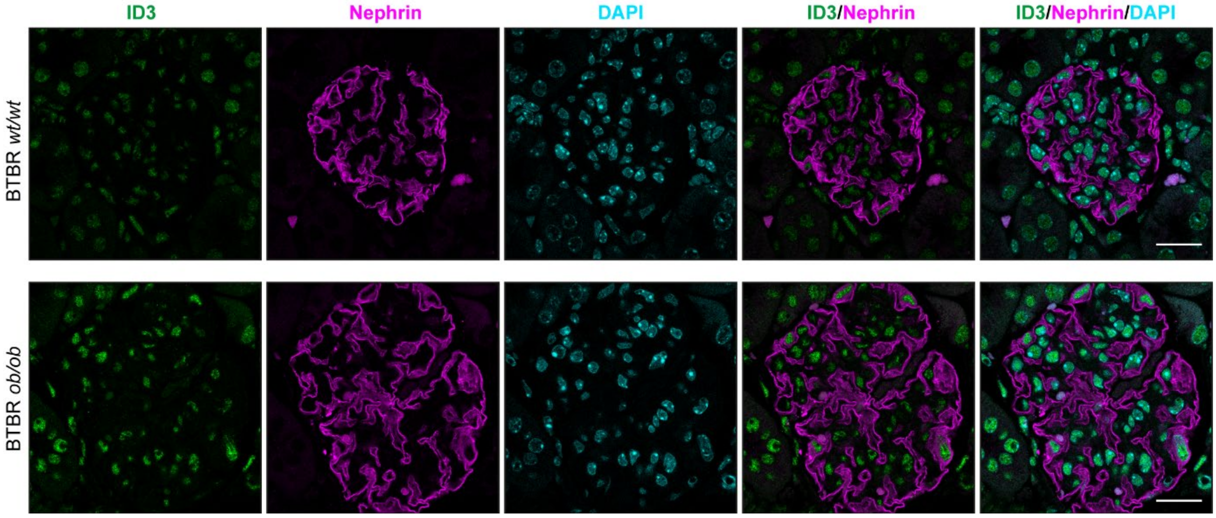

Figure S5. Co-staining for ID3 and glomerular endothelial cells or podocytes in BTBR *wt/wt* and *ob/ob* animals. (**A**) Kidney sections of BTBR *ob/ob* and control (BTBR *wt/wt*) mice were co-stained using immunofluorescence for ID3 (green), CD31 as an endothelial cells marker (magenta) and DAPI to visualise nuclei (cyan). The ID3 and CD31 channels, as well as the ID3/CD31/DAPI channels are shown as overlays on the right, as indicated above the images. (**B**) Kidney sections of BTBR *ob/ob* and control (BTBR *wt/wt*) mice were co-stained using immunofluorescence for ID3 (green), nephrin as a podocyte marker (magenta) and DAPI to visualise nuclei (cyan). The ID3 and nephrin channels, as well as the ID3/nephrin/DAPI channels are shown as overlays on the right, as indicated above the images. Shown are single optical slices from confocal stacks. Scale bars, 20 µm.

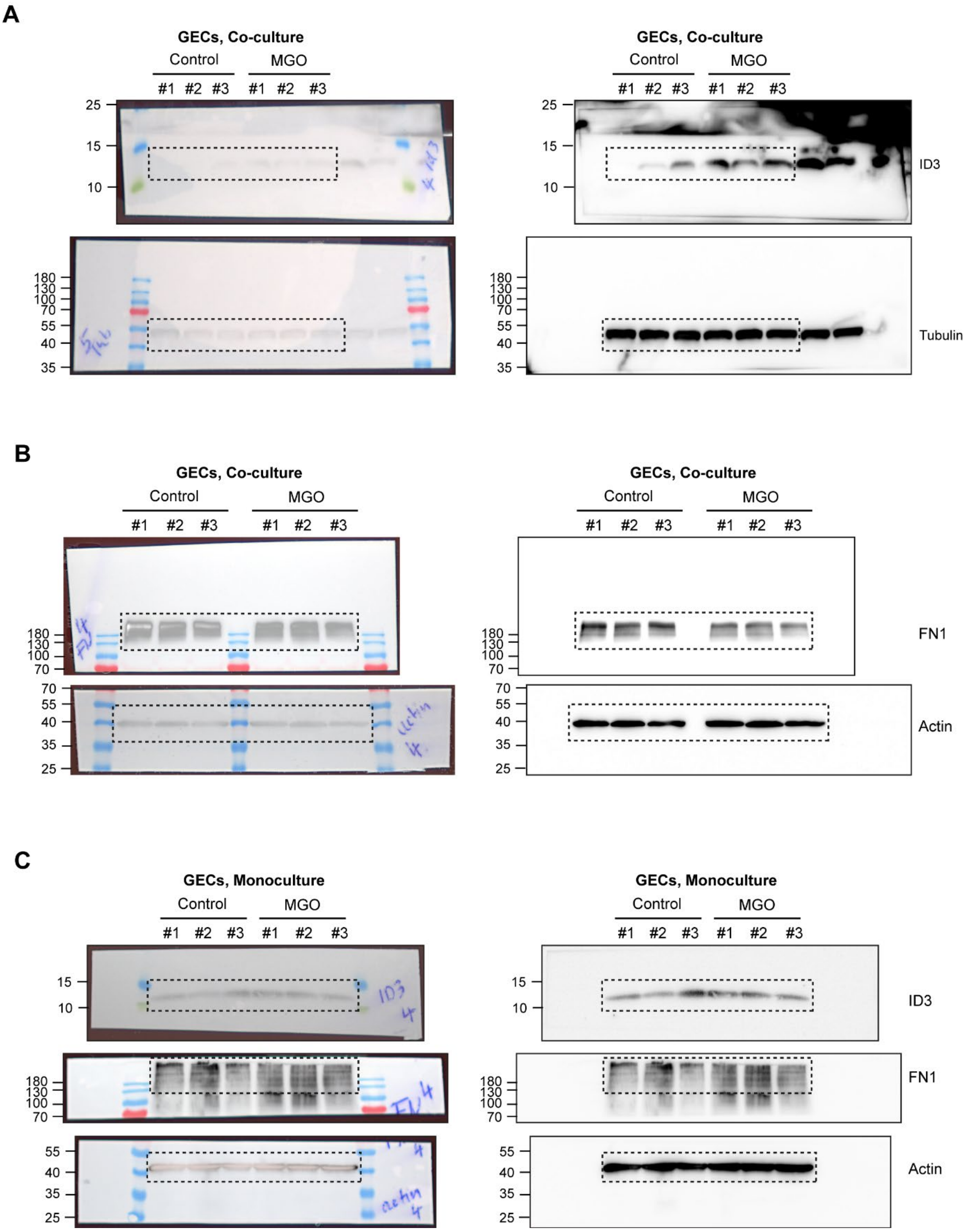

Figure S6. Original images of the full membranes for the Western blots shown in Figure S4. Panels **A**, **B** and **C** present images of the full membranes for the Western blots shown in Figure S4A, B and C, respectively. Shown on the left are photographic images of the membranes overlaid with an exposure of the chemiluminescence signal. The exposure used for assembling the corresponding panels in Figure S4 are shown to the right with the same field of view, but containing the chemiluminescence signal only. The dotted rectangles over the Western blots indicate the cropped regions used in Figure S4. The numbers to the left indicate the molecular weight of the markers that can be seen on the membranes at the respective height. The names of the antigens are indicated to the right of the Western blots.

SUPPLEMENTARY TABLES

Supplementary Table S1 (Excel). Lists of all significantly (FDR-corrected p-value <0.05) differentially expressed genes after 48 hr and 96 hr of HG exposure and after 96 hr of MGO exposure in GECs and podocytes. Each condition is shown in a separate sheet.

Supplementary Table S2 (Excel). List of all significantly (FDR-corrected p-value <0.05) differentially expressed genes that overlap between the groups shown in the Venn diagrams in Figure 1D-F and Figure S3. For each comparison, overlapping upregulated and downregulated genes are shown in separate sheets. For each gene, the $\log_2$ fold change, p-value and FDR-corrected p-value for each of the compared conditions are shown.

Primer name	Sequence
COL3A1 for	5´-CGCTCTGCTTCATCCCACTAT-3´
COL3A1 rev	5´-TGGGTTGGGGCAGTCTAATTC-3´
COL11A1 for	5´-AGGGTGAAATTGGTGAGCCG-3´
COL11A1 rev	5´-CCAAACATCCCCTGCTGTCC-3´
CSF2 for	5´-GGGAGCATGTGAATGCCATC-3´
CSF2 rev	5´-GGCTCCTGGAGGTCAAACAT-3´
CXCL1 for	5´-CCAGCTCTTCCGCTCCTCT-3´
CXCL1 rev	5´-TGGCAGCGCAGTTCAGTG-3´
CYGB for	5´-CTGGAGATGGAGCGGAGC-3´
CYGB rev	5´-GACCACCTCCAGAATGACCC-3´
CYP1A1 for	5´-CCTTCCCTGATCCTTGTGATCC-3´
CYP1A1 rev	5´-TGAGGCCCTGATTACCCAGAA-3´
EGR1 for	5´-TTCAACCCTCAGGCGGACA-3´
EGR1 rev	5´-CCAGCACCTTCTCGTTGTTC-3´
FBN1 for	5´-TCAATGGAGGAAGGTGTGTG-3´
FBN1 rev	5´-AAACATGGGCCTGTCCTGTA-3´
FN1 for	5´-AGGGGGTCAGTCCTACAAGA-3´
FN1 rev	5´-CCACGTTTCTCCGACCACAT-3´
ID1 for	5´-GGTAAACGTGCTGCTCTACG-3´
ID1 rev	5´-TTCAGCTCCAACTGAAGGTCC-3´
ID3 for	5´-GAGCTTTTGCCACTGACTCG-3´
ID3 rev	5´-TTCACAGTCCTTCGCTCCTG-3´
IL24 for	5´-ACACAGGCGGTTTCTGCTATT-3´
IL24 rev	5´-AGAATGTCCACTTCCCCAAGG-3´
ITGB6 for	5´-AGCAAACTAGCAGGCATCGT-3´
ITGB6 rev	5´-ACTTGTTCTTGGGTTACAGCGA-3´
NR4A1 for	5´-GGACAACGCTTCATGCCAGCAT-3´
NR4A1 rev	5´-CCTTGTTAGCCAGGCAGATGTAC-3´

Supplementary Table S3. Forward and reverse primer sequences used for qPCR.
